# Hyaluronan as a Prominent Biomolecule with Numerous Applications in Medicine

**DOI:** 10.3390/ijms22137077

**Published:** 2021-06-30

**Authors:** Katarína Valachová, Ladislav Šoltés

**Affiliations:** Centre of Experimental Medicine, Institute of Experimental Pharmacology and Toxicology, Slovak Academy of Sciences, Dúbravská cesta 9, 84104 Bratislava, Slovakia; ladislav.soltes@savba.sk

**Keywords:** gene delivery, hyaluronic acid, medicinal applications of hyaluronan, skin wound treatment, tissue engineering

## Abstract

Hyaluronan (HA) is a natural glycosaminoglycan present in many tissues of all vertebrates. HA has various biological functions, which are dependent on its molar mass. High-molar-mass HA has anti-angiogenic, immunosuppressive and anti-inflammatory properties, while low-molar-mass HA has opposite effects. HA has also antioxidative properties, however on the other hand it can be readily degraded by reactive oxygen species. For many years it has been used in treatment of osteoarthritis, cosmetics and in ophthalmology. In the last years there has been a growing interest of HA to also be applied in other fields of medicine such as skin wound healing, tissue engineering, dentistry and gene delivery. In this review we summarize information on modes of HA administration, properties and effects of HA in various fields of medicine including recent progress in the investigation of HA.

## 1. Introduction

**Figure 1:**
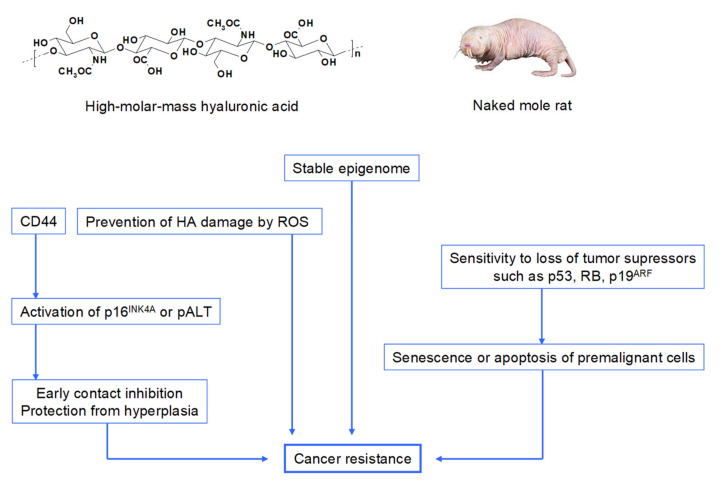
Anticancer mechanisms in the naked mole rat. HMM HA interacts with CD44 receptors and promotes early contact inhibition of naked mole rat fibroblasts via activation of p16^INK4A^ (a protein involved in regulation of the cell cycle) or the naked mole rat specific product of the *Cdkn2* gene locus, pALT. Early contact inhibition provides protection from cancer by arresting the cell cycle at a low cell density and preventing hyperplasia. HMM HA may also provide protection from metastasis by maintaining a stronger ECM. HMM HA also acts as an antioxidant, thereby reduces ROS-induced damage to nucleic acids and proteins. Furthermore, this animal has a unique ability to sense the loss of a single tumor suppressor such as p53, RB or p19^ARF^ and undergoes apoptosis or senescence (adapted from Seluanov et al. [22]).

Hyaluronan or hyaluronic acid (HA, Figure 1) is a prominent high-molar-mass linear glycosaminoglycan found in the extracellular matrix (ECM) reaching a size of up to 8 MDa. It is ubiquitous, but is especially prominent in tissues undergoing rapid growth and repair. The polymer has the structure of poly[(1→3)-2-acetamido-2-deoxy-β-d-glucose-(1→4)-β-d-glucopyranosyluronic acid)]. It has one carboxyl group per disaccharide repeating unit, and therefore, in aqueous solution, HA behaves as a polyelectrolyte with a negative charge [1]. HA is highly hygroscopic, tightly binding 15 water molecules per each disaccharide unit. Furthermore, aqueous HA solutions show a very high and shear dependent viscoelasticity, resulting in the role of HA as an extracellular lubricant [2]. HA molecules chelate iron and copper ions, which are required in the Fenton reaction. In the absence of these ions, hydroxyl radicals, as the most deleterious reactive oxygen species (ROS), cannot be formed [3,4]. Moreover, HA can also neutralize ROS outside the leukocytes and protect neighboring cells [4].

HA is widely distributed in both prokaryotic and eukaryotic cells. Adult humans contain about 12–15 g of HA, most of which occurs in skin, vitreous body of the eye, umbilical cord, synovial fluid of articular joints, intervertebral disks, embryonic mesenchymal tissues, but is also present in other tissues such as heart valves, lungs, tendon sheaths, bursas aorta, and prostate and is essential in the fertilization process [2,5,6]. HA is also highly expressed in the glycocalyx—a pericellular coating of most cells—and is particularly present on the apical surface of endothelial cells [5] and is well detected intracellularly, where it associates with the mitotic spindles, microtubules, and the receptor for HA-mediated motility (RHAMM) [7]. Synthesis of HA in skin is carried out by three HA synthases, HAS1, HAS2, and HAS3, that incorporate uridine diphosphate sugars into the non-reducing end of the growing sugar chains, which are directly extruded into the ECM [2,5,8]. In vivo degradation of HA under inflammatory and oxidative conditions is carried out through enzymatic degradation with hyaluronidases or by reactions with reactive oxygen and nitrogen species [9,10,11].

High molar mass HA (HMM HA) possesses anti-inflammatory, anti-proliferative, anti-angiogenic and immunosuppressive properties. Due to the latter effects, HA binds fibrinogen and controls the levels of inflammatory cytokines and the migration of stem cells [1,12,13,14]. Generally, HMM HA inhibits cell differentiation and promotes cell proliferation, participates in tissue regeneration, wound healing, epithelial integrity and embryogenesis, plasma protein distribution, matrix structuring and provides water homeostasis [15,16]. Moreover, HMM HA can be also inhaled, which has been used for several years in Europe with a remarkably good safety profile. Treatment with HMM HA can also inhibit inflammatory response in several disease models, e.g., HMM HA attenuated inflammation and lung injury in a sepsis model of ventilated rats [6]. HMM HA also has antioxidant effects, which link with the attenuation of DNA damage in human leukocytes during oxidative burst [3,4]. HA of molar mass in a range of 1000–5000 kDa is known for hygroscopicity and viscoelasticity, modulates tissue hydration and osmotic balance [12,13].

In the last years there has been a growing interest in exploring a rodent named the naked mole rat (*Heterocephalus glaber*, Figure 1), which lives in arid regions of East Africa. Compared to other rodents naked mole rats differ markedly in that these animals are eusocial, cold-blooded and have a single breeding female within a colony. Moreover, they have lower body temperature, they are resistant to hypoxia, hypercapnia, high oxidative stress and neurodegenerative diseases [17]. The lifespan of the naked mole rat is up to 30 years [18]. In comparison, a maximum lifespan of a similarly sized house mouse is 4 years. In addition to their longevity, naked mole rats show an unusual resistance to cancer, which is related to the fact that the fibroblasts of this animal secrete extremely HMM HA (6–12 MDa) [2,8], which is larger than HA produced in mice (0.5–3 MDa) [18] or humans (6–8 MDa [1]). Such HMM HA accumulates abundantly in naked mole-rat tissues owing to the decreased activity of HA-degrading enzymes and HAS2, which is overexpressed in their skin fibroblasts. Furthermore, the naked mole-rat cells are more sensitive to HA signaling, as they have a higher affinity to HA compared to mouse or human cells [2,18]. Such HMM HA shields the CD44 receptor from interaction with other proteins leading to distinct downstream signaling characterized by the cytoprotective effect, suppression of apoptosis. It has anticancer properties by limiting cell proliferation and metastases and may ultimately promote longevity by protecting tissues from stress. For this reason, the formed HA is suggested to be responsible for longevity of naked mole rats [8,17,18,19,20,21]. The anticancer mechanism of the naked mole rat is displayed in Figure 1 (adapted from Seluanov et al. [22]).

Unlike HMM HA, low-molar-mass (LMM) HA has immunostimulatory and angiogenic properties, thereby it induces proinflammatory cytokines, chemokines and growth factors and stimulates extracellular matrix remodeling [12,13,23]. Interestingly, HA oligosaccharides of size 4–16 mers have been shown to both stimulate and inhibit inflammatory responses depending on cell type and diseases. They have been shown to stimulate proinflammatory effects in synovial fibroblasts, promote angiogenesis during wound healing, enhance cell adhesion [7] and are supposed to act as endogenous danger signals [1].

Since its discovery, the biological functions of HA have been thoroughly investigated. For example, HA is involved in healing wounds, tissue repair and regeneration, organization of the ECM, lubrication of the joints, regulation of the cell adhesion and motility through receptors that interact with the cytoskeleton, angiogenesis by mediating cell proliferation, cell differentiation and cell migration [24]. Furthermore, HA also has applications in regenerating tissues, cosmetics, optical surgery and has immunomodulatory, anticancer and anti-proliferative properties [25,26].

The aim of this review was to summarize well-known as well as new information on hyaluronan in the treatment of diseases such as arthritic and eye diseases, applications of hyaluronan in cosmetics, dentistry, gene delivery, skin wound healing and tissue engineering.

## 2. Medicinal Applications of Hyaluronan

### 2.1. Treatment of Osteoarthritis

In the synovial joint, HA plays an important role in the protection of articular cartilage and the transport of nutrients to cartilage and is responsible for the viscoelastic properties of synovial fluid (SF) [7,27]. It could also display anti-inflammatory activities, affect proteoglycan synthesis, act as a barrier against catabolic substances on the cartilage surface and suppress the production of cartilage degrading enzymes via binding to CD44 [11,28]. HA is also complexed with lubricin, a glycoprotein, to form a network that forms a boundary lubricant decreasing friction force and greatly reducing wear damage on rubbing/shearing surfaces [28]. As normal SF is free of hyaluronidases, it has been inferred that ROS are involved in a self-perpetuating process of HA catabolism within the joint. This process is considered responsible for the short 12 h half-life of native HA macromolecules in SF [29].

HA in the SF binds to chondrocytes via the CD44 receptor, supporting a role for HA in healthy cartilage. Pretreatment of chondrocytes with HA prevents mitochondrial DNA damage, enhances mitochondrial DNA repair capacity and cell viability, preserves ATP levels and ameliorates apoptosis [30]. In addition, HA suppresses H_2_O_2_-induced cell death in human chondrocytes through an intracellular signaling pathway [4,26].

For articular cartilage, a milieu with a low level of oxygen is necessary. Peroxidation of HA requires a large amount of molecular oxygen, thus the tension of oxygen in the joint is maintained at physiologically critical level. There is a change in physical activity during day and night, with periods of joint hyperemia and ischemia, respectively. Elevated level of oxygen and the resulting oxidative stress would lead to chondrocyte dysfunction and cartilage damage [31]. The authors postulated a possible mechanism for the removal of oxygen excess. HMM HA turnover in SF utilizes peroxidative degradation, during which oxygen is massively consumed. The peroxidation itself may be initiated by H_2_O_2_, which is produced by chondrocyte mitochondria, that can diffuse into the SF. The resulting decrease in available oxygen down-regulates HA peroxidation. This in turn prevents excessive oxygen consumption. It appears that ROS may be components of normal physiology, particularly in the SF of joints and articular cartilage [31]. There are several studies supporting the fact that HA reduces levels of ROS and also protects chondrocytes from the adverse consequences of exposure to these molecules [28].

Of more than 100 arthritic diseases, osteoarthritis (OA) and rheumatoid arthritis (RA) are the most prevalent ones affecting the elderly population [32]. OA is a complex degenerative disorder of unknown etiology that affects many different joints, especially knees, hips, hands and facet joints. This disease is characterized by morphological, biochemical, molecular and biomechanical changes in both cells and ECM, which lead to softening, fibrillation, ulceration, loss of articular cartilage, synovial inflammation, sclerosis of subchondral bone, adjacent connective tissues, pain and stiffness of the affected joint, formation of osteophytes and subchondral cysts [27,32,33,34]. Decreased HA synthesis, increased HA degradation and elevated oxidative stress lead to a decrease in both concentration and average molar mass of the HA present in the synovium [7,27,28].

Unlike OA, RA is classified as a systemic inflammatory disease, in which pain of one or more joints is usually accompanied by degenerative changes in other organs, such as lungs, heart and blood vessels, however, the etiology and pathogenesis of RA has not been elucidated yet. In acute phases, neutrophils are accumulated in the patient’s synovial fluid. These cells alter the oxidative homeostasis, and their products, especially ROS, can contribute to the destruction of joint structures to such an extent that it is not functional any more. The damaged tissues are considered as foreign, and subsequently autoimmune reactions make the disease worse [32]. Like in OA, there are reduced HA concentrations in synovial fluids from RA patients [11,35].

For the treatment of pain associated with OA intraarticular (IA) HA therapy is used, which is known as viscosupplementation [17,36]. There is a growing body of preclinical and clinical data, which suggests that IA administration of HA also has disease-modifying activity, which include: (1) promotion of healing and repair of cartilage; (2) maintenance of chondrocyte vitality (decreased apoptosis); (3) inhibition of destruction of chondrocytes; (4) stimulation of synthesis of articular cartilage matrix components (e.g., collagen, proteoglycans, including endogenous HA, hyaladherins); (5) stimulation of chondrocyte growth and metabolism; (6) inhibition of matrix-destructive inflammatory processes; and (7) inhibition of expression and activity of chondrodegradative enzymes, e.g., metalloproteinases [4,30,36].

The efficacy and tolerability of IA HA for the treatment of pain associated with OA of the knee have been demonstrated in several clinical trials. Three to five Hylan G-F 20 sodium hyaluronate injections can provide relief of knee pain from OA for up to 6 months. IA injections of HA are generally well tolerated, with a low incidence of local adverse OA is characterized by a slow degradation of cartilage over several years [27]. Studies have also shown that IA injection of HA in patients with OA increases endogenous HA production [28]. Yoshioka et al. [37] showed that polyhydroxylated C_60_ fullerenes, especially C_60_(OH)_10_ C_60_(OH)_24_ and C_60_(OH)_26_, may protect against OA related factor-mediated downregulation of osteoarthritic chondrocyte activities.

HA can be also introduced into the body orally, where the body absorbs the HMM HA as a decomposed 2–6 membered polysaccharide. One proposed mechanism of action shows that ingested HA binds to Toll-like receptor-4 and promotes the expressions of interleukin-10 and cytokine signaling, which both lead to reduced inflammation in arthritis [38]. Reports on oral HA clinical trials performed by Oe et al. [39] found that in patients, highly pure HA had a beneficial effect on knee pain compared to the placebo. During the 12-month study when 30 patients took an oral HA, no statistically significant negative side effects were seen.

Many trials have been performed to investigate the role of IA injection of HA in alleviating the symptoms in hip OA. A randomized clinical trial compared the effects of HA injection with platelet rich plasma (PRP) injection, where Battaglia et al. [40] demonstrated that administration of HA rather than PRP is more appropriate in patients with symptomatic hip OA, where relief of pain and functional improvement was seen. In another study Vad et al. [41] reported that three Hylan G-F injections would be safe and an essential option for mild and moderate hip OA to reach rapid pain relief. However, no efficacy was shown for patients with severe hip OA. In a pilot study, the effectiveness of the IA injection of Euflexxa was examined (1% sodium non-crosslinked of molar mass 2.4–3.6 MDa) in 22 patients with OA of the subtalar joint [42,43]. Tamura et al. [44] showed that IA injection of DK226 (a novel conjugate composed of HA and methotrexate) showed similar anti-arthritic effects as oral methotrexate—a drug for treatment of RA and OA of the knee. In another study, nanoparticles composed of poly(d,l-lactic acid) or poly(d,l-lactic-co-glycolic acid) covered by chemically esterified amphiphilic HA are being considered as drug carriers for treatment of OA [45].

Preparations of injectable HA differ in many parameters, including source (animal or bacteria), mean molar mass in a range of 500–6000 kDa, distribution of molar mass, molecular structure (linear, crosslinked or both), method of crosslinking, concentration in a range of 0.8–30 mg/mL and volume of injection in a range of 0.5–6.0 mL. A traditional source of HA for many years was a rooster comb. Currently, a modified bacterial source *Streptococcus zooepidemicus* is used as the main source as it is associated with lower costs and less side effects [46].

The pain-relieving benefit of IA HA generally persists for considerably longer than its half-life within the injected joint, which has been estimated to be as short as 18–24 h in animal studies. For example, clinical efficacy in randomized, controlled trials has been demonstrated to last for at least 26 weeks for Hyalgan (sodium hyaluronate, average molar mass 500–730 kDa, Fidia Farmaceutici S.p.A., Abano Terme, Italy) and may last as long as a year or more in some patients. Similarly, Synvisc (Hylan G-F 20, average molar mass 6000 kDa, Genzyme Biosurgery, Cambridge, MA, USA), Supartz (sodium hyaluronate, average molar mass 630–1120 kDa, Seikagaku Corporation, Tokyo, Japan) and Orthovisc (sodium hyaluronate, average molar mass 1900–3000 kDa, Anika Therapeutics, Bedford, MA, USA) and other products such as Gel-One (Zimmer, Warsaw, IN, USA); Euflexxa (Savient, Newport Beach, CA, USA); Monovisc (Anika Therapeutics, USA) and Gel-Syn (Institut Biochimique SA, Lugano, Switzerland) have also demonstrated months of pain relief [36,38]. A novel HA-based hydrogel, Hymovis (Fidia Farmaceutici S.p.A., Abano Terme, Italy) was injected IA to patients in 2 doses at concentration 24 mg/3 mL within 1 week interval. This hydrogel was effective particularly after 6 months post-injection and provided a therapeutic tool in the treatment of moderate and severe OA [47]. Hyalubrix/HyalOne formulation of HMM HA had an enhanced safety profile and was effective after the first injection, significantly reduced consumption of nonsteroidal antirheumatoid drugs and can be used for repeated therapy cycles over more years as a conservative therapy to delay replacement of hips [48]. Viscosupplementation with IA Sinovial^®^ injections (molar mass 800–1200 kDa, 16 mg/2 mL) relieves pain and improves function in OA of the knee and other joints including the carpometacarpal joint of the thumb and the shoulder [49]. In another study the authors showed that a single IA injection of 3 mL IA HA solution Arthrum in a dose 75 mg (>2 MDa), (LCA Pharmaceutical, Chartres, France) within 6 months was effective in clinical trials [50]. JTA-004 is a new preparation for IA treatment of knee OA. It is a mixture of plasma proteins and HA. In a phase II study, which involved 164 patients, JTA-004 showed improved pain relief within 3 and 6 months compared to Hylan G-F 20—the market leader in OA treatment worldwide. Currently it is in a phase III clinical study [51]. Furthermore, to minimize the number of IA injections, a smart approach has been patented [52]; a mixture of two self-associating HA derivatives is applied into the joint where a viscoelastic gel is formed [53].

There are several papers documenting treatment of temporomandibular joint (TMJ) with HA. HA in a traumatic, degenerative or inflammatory TMJ improves its function and decreases pain, due to its mechanical and metabolic properties, which involve providing nutrition to avascular areas of the condylar cartilage and disc [6]. It is demonstrated that biodegradable mesoporous silica nanoparticles successfully deliver the enzyme HAS2, into synoviocytes from the TMJ and generate endogenous HA of HMM. In a rat TMJ OA inflammation model, this strategy promotes endogenous HA production and inhibits the synovial inflammation of OA for more than 3 weeks with administration of one injection [54]. In another paper the results showed that HA and PRP injection provided a marked improvement in patients with TMJ disorders compared to the HA injection alone [55]. Zhu et al. [56] published new and potential insights into treatments of TMJ disorders using poly(d,l-lactic-co-glycolic acid) microspheres loaded with HA and parecoxib as a successful drug delivery system. Other recorded uses of HA are procedures of maxillofacial surgery, orthopedics and orthognathic surgery [6].

HA also occurs in bones, whereas natural bone is mainly composed of HA crystals deposited in a collagen matrix and can exhibit either a trabecular or a dense morphology [57]. HA also promotes bone regeneration, which is carried out by regulating cell activity and the release of biological factors. HA bound with CD44 receptor can be incorporated into the cytoplasm of osteoprogenitor cells to regulate their migration. Moreover, insufficient vascularization of bone substitutes often results in poor bone regeneration. The fragments of HA can enhance angiogenesis, which is essential in the process of bone formation, via RHAMM-mediated signaling pathways in epithelial cells [58].

### 2.2. Cosmetics

In skin, where 50% of HA is present [59,60], HA maintains dermal volume and viscoelasticity, however in dermis HA has a half-life of ca. 1 day and in epidermis as little as 2–3 h. For this reason, HA is not appropriate to be used as a dermal filler. To remove this hindrance, the crosslinking of HA is necessary, which stabilizes HA molecules in tissues, and by the interaction with other matrix proteins, such as collagen, complex supramolecular structures are formed [9,10]. Since 1996, HA has been launched onto the market in Europe. To date, various companies produce HA. Biomatrix (NJ, USA) produces a rooster comb-derived HA. Q-Med AB (Uppsala, Sweden) and LEA-DERM (Paris, France) are the main companies to provide HA by bacterial fermentation (the strain of *Streptococci*). No skin testing of HA is necessary before injecting because HA is a biodegradable agent. The HA is not used to fill in but rather to hydrate and finally to rejuvenate the skin using injection into the superficial dermis and epidermis. This procedure must be repeated in intervals of a few weeks or months. Although currently HA is the safest filler agent in cosmetic indications, some side effects can appear, however, most of them are not severe and will disappear when the product is degraded [61]. HA dermal fillers have frequently been used for facial soft tissue augmentation because of their longevity, ease of use and low immunogenicity. Stabilized HA gels can stimulate collagen synthesis and inhibit collagen degradation, which can further contribute to their long-lasting effects. Due to its nonanimal source, HA also has a minimal risk of inducing previously reported hypersensitivity reactions. In cases of facial lines resulting from the loss of volume associated with aging, injectable fillers, which efface and support the static rhytides, are the most suitable treatment. HA fillers from different manufacturers vary in characteristics such as total HA concentration, modulus, size of particles, degree and percentage of HA crosslinking, the amount of unmodified HA present and extrusion force [59].

The HA fillers injections are the second most-frequently carried out aesthetic medicine procedures in the world after botulinum toxin injections [62]. The most commonly used dermal fillers for facial rejuvenation are HA-based hydrogels, produced by crosslinking of HA with 1,4-butandiol diglycidyl ether. The findings of La Gatta et al. [63] were the first wide evaluation of features for the volumetric class of HA-fillers and included the first data on biological effects on human dermal fibroblasts and their resistance to degradation by ROS. In another study, a positive filling effect of Fillerina^®^ on decreasing the clinical signs of aging skin and on improving the face volume was shown [64]. Mannitol as a scavenger of ROS and a protector from HA degradation was incorporated into a mesoderm therapy product Juvederm Hydrate^®^, which comprises 13.5 mg/g non-crosslinked HA and 0.9% mannitol. Mannitol is also incorporated into the products now marketed as Etermis^®^ (Merz Pharmaceuticals GmbH, Frankfurt am Main, Germany). Glytone 1^®^ is a high molar mass (3.4 MDa), uncrosslinked HA-based mesotherapy product (14 mg/mL) which contains mannitol (34 mg/mL) and glycerine (3 mg/mL) and is used to improve quality of skin [9]. In another study, Tan et al. [65] reported the preparation of the product M89 containing 89% of Vichy mineralizing water and HA, which significantly improved skin signs and symptoms after 4 weeks of continued use in 1630 individuals with mean age 41.1 ± 11.3 years. M89 served as adjunct to conventional therapy. Moreover, individuals who have recently undergone esthetic procedures M89 observed a satisfying skin recovery.

The first HA-based filler approved for use in the United States was Restylane, followed by Restylane-L, Lyft, and Silk. They are concentrated with 20 mg/mL of HA and have 1 volume% of crosslinking. In 2016, Galderma introduced two new products, Refyne and Defyne. Juvederm (Allergan) is another line of HA fillers first gaining FDA approval in 2006. By this process a variable and highly crosslinked HA from 100 volume% of high molar mass chains with 24 mg/mL of HA is produced. Juvederm Volux is another new product for deep soft-tissue augmentation. It contains 25 mg/mL HA concentration and is under investigation for chin and jawline augmentation. Belotero Balance (Merz Aesthetics, Greensboro, NC, USA) is another HA filler approved in 2011 with HA content 22.5 mg/mL. Currently, a new HA filler marketed as Teosyal (Teoxane, UK) is under study in the USA. It consists of monophasic HA at a concentration of 23 mg/mL. In this product, protein and bacterial endotoxins are reduced, which resulted in less hypersensitivity reactions [66]. Other brand names of products containing HA include: Captique, Esthélis, Elevess, Hylaform, Perlane, Prevelle and Puragen [67], Dermalive, Hyalaform, Ac Hyal, Hylan Rofilan Gel and Reviderm [59]. A novel HA preparation, Profhilo^®^ (IBSA Farmaceutici Italia Srl, Italy), is based on stabilized, cooperative, hybrid complexes, produced with NAHYCO hybrid technology, an innovative patented thermal production process, by which HA is stabilized without using any crosslinking agents. Concentration of HA is 32 mg/mL, which is the highest among HA preparations. Further, the preparation has optimal tissue diffusion and high manageability, which enables a multilevel dynamic tissue remodeling [68].

### 2.3. Skin Wound Healing

There are five phases of skin wound healing, namely, homeostasis, inflammation, proliferation, remodelation and granulation [60]. During the early stages of wound healing, HA serves as a temporary structure primarily because of its large molecular size. Its unique structure provides the diffusion of nutrients and waste products in the injury site. HA directly affects the proliferation and migration of keratinocytes. Several important functions associated with HA during the wound healing process stem from the CD44 receptor. In the wound environment, CD44 is responsible for the internalization of HA degradation products and is an essential receptor during the inflammatory response. Furthermore, CD44 is responsible for the recruitment of fibroblasts into the wound area from surrounding tissue. Another receptor known as RHAMM helps the healing response. Intracellularly, RHAMM, along with cytoskeletal proteins, activate several protein kinases which stimulate cellular movement. These systems are essential to tissue repair and the inflammation processes. RHAMM is also highly expressed on the surface of fibroblasts and has shown to directly stimulate proliferation of fibroblasts in vivo.

**Figure 2:**
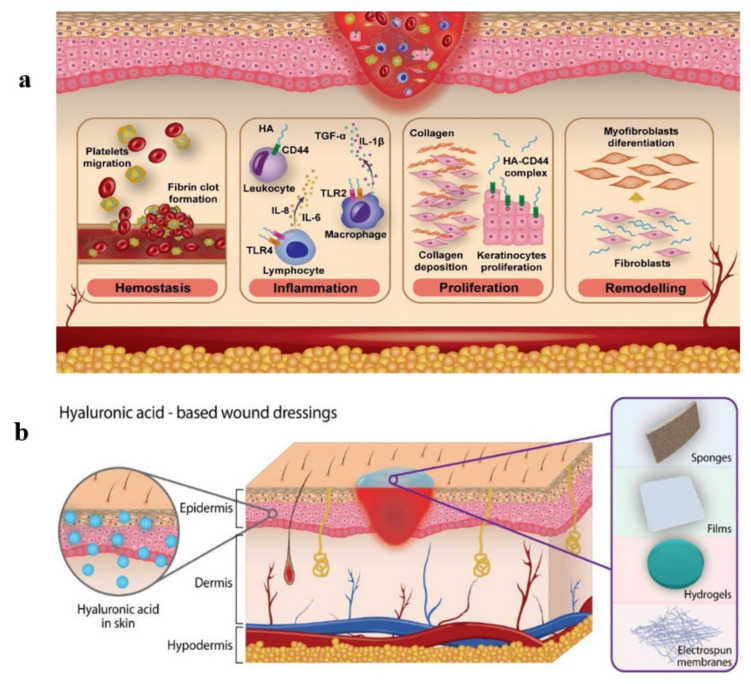
(**a**) Role of HA in phases of skin wound healing; (**b**) various skin wound dressings based on HA, adapted from Graça et al. [73].

In the initial stage of wound healing, i.e., homeostasis, HMM HA (~2000 kDa) accumulates in the extracellular matrix and binds to fibrinogen to form a clot. Thereafter, in the inflammatory phase, HMM HA is fragmented into LMM HA (80–800 kDa) by hyaluronidase for subsequent usage in healing [69]. A major function of HA is the modulation of inflammatory cells and dermal fibroblast activities, e.g., cellular migration, proinflammatory cytokine synthesis and the phagocytosis of invading microbes. LMM HA binds to TNFa TLR4 to induce inflammatory responses stimulating IL-6, IL1β [70]. During this phase HA regulates the hydration level of the tissue. HA induces and promotes angiogenesis, production of ECM, enhanced production and differentiation of fibroblasts and keratinocytes [71]. The levels of HA synthesized by both fibroblasts and keratinocytes are elevated during re-epithelialization. HA binds to cells via receptors such as CD44, RHAMM and ICAM, whereas CD44 is recognized as the major receptor of HA, whose interaction induces many physiological events, such as cell migration and proliferation [70]. Angiogenesis, an important aspect for wound repair, is induced by LMM HA fragments (8–16 oligomers) [72]. It is well known that one of the hallmark features of chronic wounds is the prolonged inflammatory phase caused by the production of ROS and matrix degrading enzymes such as proteases and matrix metalloproteinases. Therefore, the most important mechanism of HA in tissue regeneration is its ability to serve as a free radical scavenger, which happens in the process of granulation of the tissue [71]. It was found that LMM HA prevented damage of the granulation tissue by ROS and increased the self-defense of skin epithelium by inducing various skin-repair-related genes during the healing process [69]. Figure 2 displays the role of HA in different phases of skin wound healing and types of wound dressings based on HA.

Wound dressings are traditionally used to protect the wound site from contamination, but they can be exploited as materials to deliver bioactive molecules to wound sites. The use of topical bioactive agents in the form of solutions, creams and ointments for drug delivery to the wound is not very effective as they rapidly absorb fluid, and lose their rheological characteristics and become mobile. For this reason, the use of solid wound dressings is preferred in case of exudative wounds as they provide better exudate management and prolonged residence at the wound site. Unlike traditional dressings such as gauze and cotton wool that do not actively participate in the process of wound healing, advanced dressings are designed to have their own biological activity or may release incorporated bioactive constituents such as drugs from the wound dressing. The incorporated drugs can play an active role in wound healing either directly as cleansing or debriding agents for removing necrotic tissue, or indirectly as antimicrobial drugs, which prevent or treat infection and thus regenerate the tissue [74].

Exogenously applied HA has been shown to improve healing of a variety of tissues. A new approach for fabrication of HA into three-dimensional shapes such as strands has been reported, with improved residence time and cell adhesion. It has been hypothesized that inoculation of an implanted matrix with exologous cells would promote healing by supplementing the immigration of cells from surrounding tissues. It was shown that modified 3D HA strands provided an effective surface-to-volume ratio for cells to populate, remarkably enhanced wound healing and reduced scar formation [25]. HA is used in dermatological treatment as a matrix for autologous skin transplants [75].

The biomaterials used for wound healing should be biocompatible, biodegradable, non-immunogenic, non-toxic, non-inflammatory and mechanically applicable [13]. The primary function of a wound dressing is to stop bleeding and absorb exudates, whereas additional biocomponents such as adhesive proteins and growth factors can augment repair by means of their inherent wound healing properties [76]. They are generally preferred in full-thickness lesions, e.g., traumas, surgical wounds and chronic ulcers and have been shown to minimize hypertrophic scarring, contractures and increase scar elasticity in acute burn wounds [12]. HA along with cellulose, chitosan, collagen and alginate, are the most commonly used biopolymers that currently prevail in wound treatment [77]. HA-based wound dressings can be in a form encompassing hydrogels, films, scaffolds, foams, topical formulations and nanoformulations [78].

HA serves as a valuable material to create hydrogels that assist in healing skin wounds. However, native HA is not useful and must first be crosslinked in order to provide stability and improve functionality of gels. To crosslink HA, different methods such as polyvalent hydrazide crosslinking, water-soluble carbodiimide crosslinking, disulfide crosslinking, divinyl sulfone crosslinking and photo-crosslinking through glycidyl methacrylate-HA conjugation have been used. Crosslinked HA hydrogels have several applications in the field of bioengineering such as molecule delivery, cell delivery, cartilage tissue engineering and development of micro-device systems [40].

In general, the aims to modify HA are as follows:

- To adjust properties of HA including viscosity, elasticity and hydrophilicity,
- To elevate HA resistance to degradation, and thereby enhancing its residence time and the duration of its effects,
- To have HA in a form of a gel or hard textured scaffold with specific pore and particle sizes for specific cellular functions (such as cell adherence or migration),
- To produce HA-drug micelles and conjugates for sustained or targeted drug release,
- To bind HA to natural or synthetic compounds such as natural polymers, proteins, synthetic polymers, drugs and liposomes to obtain required physicochemical or therapeutic properties [79].

Hydrogels are networks of hydrophilic polymer chains that form 3D structures. These structures swell in response to water and other conditions like pH and temperature, but can still maintain their structure. Most HA-based hydrogels also contain other components such as therapeutic drugs and cytokines [60]. Improved hydrogels are continuously being made with the aim to maximize the effect of treatment. One such is called Gel-One, which is composed of a product called Gel-200, a crosslinked HA hydrogel. This product was first shown to have chondroprotective, anti-inflammatory effects and long-lasting analgetic effects in OA mouse models. Another new product, Hyajoint Plus, was shown to produce a longer lasting and stronger effect on pain than compared to Synvisc-one, which is currently used by many physicians in IA injections. Furthermore, the product Cingal combined HA hydrogels with triamcinolone hexacetonide, a long-acting corticosteroid previously shown to help treat OA. A clinical trial showed that Cingal provided immediate and long-term relief of OA-related pain, stiffness, and efficacy for 26 weeks when compared to saline. Additionally, a new product named Cartistem paired HA hydrogels with human umbilical cord blood-derived mesenchymal stem cells has been used. In a clinical trial, the product showed maturing repair tissue within 12 weeks and pain moderated for 24 weeks, both of which remained stable over 7 years [40]. Fibrin, microporous HA, and composite hydrogels were utilized to examine the effect of conductive scaffolds in the wound healing process. Composite hydrogels were paired with plasmin-degradable VEGF nanocapsules to investigate its impact as an inductive composite hydrogel on tissue repair. By 7 days, wound healing and vessel maturation within the newly formed tissue was significantly improved by the inclusion of porous scaffold architecture and VEGF nanocapsules [80].

Some biomedical scaffolds require the use of small fibers in a range of micrometers to nanometers and to form a complete scaffold. Electrospinning is a very simple and effective technique that allows the manufacturing of such fibers. Although it has been accomplished, electrospinning of HA is still a difficult process due to its high viscosity and surface tension. Some efforts have already been made to make electrospinning HA easier by interaction with other polymers such as gelatin and collagen [60].

Nanofibers made of HA are believed to be more appropriate for wound healing than solid HA forms since the nanofibers could act as scaffolds to facilitate the migration and proliferation of cells in wounds. HA nanofibers are appropriate for drug delivery. They facilitate the release of pharmaceuticals almost immediately when in contact with moisturized environments. However, because of the rapid release and degradation, HA nanofibers are not suitable for long-term drug release implants [60].

HA can also take the form of the self-assembled nanoparticles. As HA can specifically bind to various cancer cells that overexpress the CD44 receptor, many studies have focused on the pharmaceutical applications of HA-nanoparticles as a form of drug delivery. To drugs that have been used along with HA belong doxorubicin and paclitaxel. These combinations have exhibited enhanced ability of targeting and higher efficacy to other anticancer agents. Furthermore, the HA nanoparticles have demonstrated a better accumulation at the tumor site compared to water soluble HA derivatives. These nanoparticles have also been reported to restore photo-aged skin [60].

HA-based scaffolds have also been widely used in the medical industry for various therapeutic purposes. HA scaffolds can be applied in bone tissues, in nerve and brain tissue repair as well as in cell delivery and muscle regeneration. Scaffolds are only temporary supporting structures that can help promote cell and tissue growth using biodegradable structures as hydrogels [60]. HA-based scaffolds have been studied due to their excellent biocompatibility. One non-woven fleece of 100% benzyl-esterified derivative of HA was already marked as Hyaff (Fidia Advanced Biopolymers, Turin, Italy) and its efficacy used for cultured epidermal graft (Laserskin) or dermal graft (Hyalograft 3D) was demonstrated in extensive burns. Fibroblast–keratinocyte composite based on this Laserskin membrane can produce skin equivalent, and promising in vitro results suggested its potential application for burns and chronic wounds [81]. HA-based dressings have been used for temporary coverage of large skin defects until autologous skin grafts can be applied. In a case study on a tumor resection patient with a large forehead skin defect after surgery, an HA-based dressing promoted good vascularization and granulation tissue formation. Moreover, the wound indicated no hypertrophy or scarring after healing. Such dressings have also been successfully employed for the treatment of skin lesions in pediatric purpura patients [82].

Commercially available HA-based products for wound healing on the market are Hyalofill-f, Hyalofill-R, Connettivina, Connettivina Plus, Jaloskin, Hyalomatrix and Hyalosafe. The Hyalofill family products are absorbent, soft and conformable fibrous fleece or rope composed of HYAFF, an ester of HA. Connettivina is a cream containing 10 mg/mL of hyaluronate sodium used for the treatment of skin irritations and assures a hydrated environment [12,73,83]. Jaloskin is a transparent film dressing for the treatment of superficial moderately exuding wounds. Hyalosafe is a transparent film wound dressing indicated for use in the treatment of first and second-degree burns. The use of the product in deep partial thickness burns in pediatric patients to cover the burn at recovery prior to dermabrasion has also been reported. Hyalomatrix is a bilayered, sterile, flexible and conformable wound dressing composed of HA which is non-crosslinked with outer silicone membrane used in burns, diabetic ulcers and chronic wounds [12,15,82]. Another product, Hyalomatrix PA, is a bilayered wound device that is an acellular, dermal skin substitute for the temporary coverage of partial or full-thickness wounds and functions as a 3D scaffold. These authors demonstrated that this product is appropriate to be used in wounds with exposed tendon or bone [71]. Viniferamine skin and wound care products including silicone barrier contain dipotassium glycyrrhizate, which helps protect HA from degradation [84]. Another product is Hyiodine (Contipro, Dolní Dobrouč, Czech Republic), which is a patented complex of 1.5% sodium HA, 0.15% potassium iodide and 0.1% iodine produced by bacterial fermentation, and developed for the treatment of skin wounds [85]. HA hydrogels, among others, are widely used as dermal and transdermal drug delivery systems. These innovative carrier systems were designed for the controlled release of drugs through the skin into the systemic circulation, in order to maintain consistent efficacy and reduce the dose and potential side-effects of the drugs [24]. Moreover, as a topical drug delivery system for diclofenac, an HA gel has recently been approved for the treatment of actinic keratoses [86].

In our previous studies, chitosan/HA composite membranes loaded with various drugs were fabricated. One of the loaded drugs was edaravone, which was potent in treating wounded rats [87]. Further, Tamer et al. [88] fabricated and characterized membranes comprising chitosan, HA and a mitochondrially targeted antioxidant MitoQ, where MitoQ was potent in healing of skin wounds of rats and ears of ischemic rabbits. The chitosan/HA composite membranes loaded with a thiol-based antioxidant glutathione (GSH) were shown to promote the treatment of skin wounds in rats faster than in untreated rats and rats treated only with membranes without GSH [89]. Similarly, tiopronin and captopril added to chitosan/HA membranes were potent to enhance the curing of lacerations in ischemic ears of rabbits [90]. Hassan et al. [91] published a paper where phosphatidylcholine dihydroquercetin examined in skin wounds of rats showed a beneficial effect when added to the chitosan/HA membranes. Ergothioneine, hercynine and histidine added individually to chitosan/HA membranes also played a role in faster healing of skin wounds in ischemic rabbits [92]. Soltes et al. [93], patented composite membranes containing a smart-released cytoprotectant targeting the inflamed tissue. In 2021, Valachova et al. published a review about chitosan and HA, which also involved skin wound healing [94]. Table 1 summarizes skin wound dressings prepared from HA alone or in combination with another component.

There are numerous papers reporting preparations of wound dressings composed of three composite wound dressings involving HA biopolymer, e.g., HA, polycaprolactone and encapsulating growth factor [110], HA-povidone-iodine [111], HA, curcumin, pullulan [112], cornstarch/HA/propolis film [113], silk fibroin, HA, sodium alginate composite scaffold [114], HA gel, coenzyme Q10 or vitamin E, benzocaine [115], oxidized HA, vancomycin, adipic acid dihydrazide [116], HA, gelatin, genipin and hinokitiol [117], graphene oxide/copper nanoderivatives-modified chitosan/HA [118], calcium alginate hydrogel loaded with protamine nanoparticles and HA oligosaccharides [119], nanofibrous/gel structure of chitosan-gelatin/chitosan-HA [120], poly dimethylaminoethyl acrylate-HA with *Didymocarpus pedicellatus* extract hydrogel [121], HA, gelatin and chitosan scaffold [122], HA, silk fibroin, chitosan [123], chitosan citrate/nonwoven fabrics impregnated with HA [124], chitosan-HA containing new arginine derivatives with thiazolidine-4-one scaffold [125] and HA/chitosan hydrogels with vancomycin containing poly(d,l-lactic-co-glycolic acid) microspheres [77].

### 2.4. Tissue Engineering

Tissue engineering evolved from the field of the development of biomaterials and refers to the practice of combining scaffolds, cells and biologically active molecules into functional tissues. The aim of tissue engineering is to assemble functional constructs that maintain, restore or improve damaged tissues or whole organs. Artificial skin and cartilage are examples of engineered tissues that have been approved by the FDA in the USA, however, currently their use in human patients is limited. Regenerative medicine is a broad field that includes tissue engineering but also incorporates research on self-healing, where the body uses its own systems, sometimes with help of foreign biological material to recreate cells and rebuild tissues and organs. This field continues to evolve. In addition to medical applications, non-therapeutic applications include using tissues as biosensors to detect biological or chemical agents and tissue chips that can be used to test the toxicity of experimental medications [126].

Tissues can be formed in two ways: In general, groups of cells make and secrete their own support structures, i.e., ECM. This matrix supports the cells and also transmits signaling molecules. By understanding how individual cells respond to signals, interact with their environment and are organized into tissues and organisms, researchers are capable of modulating these processes to repair damaged tissues or even create new ones. Once scaffolds are created, cells in the absence or presence of growth factors can be introduced. If the environment is appropriate, a tissue develops. In some cases, the cells, scaffolds and growth factors are all mixed together at once, allowing the tissue to self-assemble. Another method to create a new tissue is to use an existing scaffold. The cells of a donor organ are stripped and the remaining collagen scaffold is used to grow the new tissue. This process can be used to bioengineer lung, heart, kidney and liver tissue and it is a perspective to use a scaffold from a human tissue discarded during surgery and combine it with a patient’s own cells to make customized organs that would not be rejected by the immune system [126].

To date, supplemental skin grafts, cartilage, bladders, small arteries and even a full trachea have been implanted in patients, but the procedures are still experimental and very costly. More complex tissues of organs such as liver, heart and lung have been successfully recreated in the lab, however, they are a long way from being fully reproducible and ready to implant into patients. These tissues, however, can be quite useful in research, especially in drug development [126].

More recently, different types of skin constructs have been designed to mimic the ECM of the skin using components such as collagen, HA and some have skin cells incorporated into them. Technologies regarding recombinant protein production, particularly of human origin, are becoming more common with increasing presence in the research literature. Both 3D Hyalograft and Hyalomatrix are HA-derived matrices that incorporate autologous fibroblasts [127]. Three-dimensional bioprinting is a booming additive manufacturing technology that allows layer-by-layer deposition or a cell-laden material to fabricate 3D constructs with spatial control over scaffold design. This technology has been widely used in the last few years for tissue engineering and regenerative medicine applications as it allows the artificial reconstruction of the complexity of native tissues or organs [128]. Use of HA with other components for various fields of tissue engineering and their properties are summarized in Table 2.

### 2.5. Ophthalmology

Hyaluronic acid is widely used in ophthalmology due to its viscosupplementant properties and its presence in the cornea, the sclera and the vitreous body. Products used to maintain the structure of the eye’s capsule, mainly in the phacoemulsification (removal of the vitreous body) step of cataract surgery but also in other surgical procedures such as vitreoretinal surgery, anterior segment surgery, glaucoma surgery, corneal transplantation and intraocular lens implantation. Some studies have been performed to produce biocompatible materials for the vitreous body replacement after vitrectomy using a new crosslinked HA through adipic acid dihydrazide bridges [15]. Furthermore, it has been demonstrated that treatment with eye drops containing HA reduces oxidative stress in the conjunctiva of patients with dry eye disease, increases tear-film stability and reduces subjective symptoms, such as ocular irritation and burning [4]. Other functions of HA in the human eye include retaining water, thickening the tear film, decreasing the tear evaporation rate, increasing tear film breakup time, lubrication of the ocular surface during blinking and movement, maintaining corneal wettability and prevention of corneal dehydration, promotion of cornel epithelial migration, stabilization of ocular surface epithelial barrier, prevention of ROS-induced cellular damage, decreasing ocular inflammation, amelioration of toxic effects of ophthalmic solution preservatives, promotion of wound healing and epithelial repair [151].

Silvani et al. [152] prepared new artificial drops based on arabinogalactan and HA and monitored the uric acid and the ROS formation. The effect of the arabinogalactan, HA and their mixture has been studied. The arabinogalactan entails uric acid and ROS reduction by 27% and 38%, respectively; no significant reduction of uric acid or ROS has been observed after the addition of HA alone. Notably the combination of arabinogalactan and HA involves the reduction of uric acid and ROS equal to 38% and 62%, respectively. It was reported that HMM HA (1000 kDa) could protect corneal epithelial cells against oxidative damage induced by UV B, benzalkonium chloride and sodium lauryl sulfate. The authors reported for the first time that HA can protect human corneal epithelial cells against the toxicity of Cr(VI) [153]. It was reported that in patients (11 in total), a combination of HA and serum eye drops could result in a gradual, slow release of growth factors over a prolonged period of time, which has beneficial effects on the ocular surface [154].

Due to the unique anatomical structure of the eye, ocular drug delivery is a promising delivery route for the treatment of several ocular diseases, such as the ocular neovascularization that contributes to diabetic retinopathy. In the study by Apaolaza et al. [155], the combination of gold nanoparticles and low molar mass HA enhanced the stability of the whole carrier and promoted their distribution across ocular tissues and barriers to reach the retina. Moreover, analysis in vitro, ex vivo, and in ovo revealed the protective and antiangiogenic effect of gold nanoparticles as inhibitors of advanced glycation end-products-mediated retinal pigment epithelial cell death and neovascularization. The authors showed that conjugation with HA enhances gold nanoparticle stability and distribution due to a specific CD44 receptor interaction. In another study, a crosslinked, thiolated HA (CMHA-S) film for treating corneal chemical burns in male New Zealand rabbits was applied. The CMHA-S treatment resulted in significant decreases in the areas of corneal opacity during 48 h, 96 h and on day 14 postoperatively. A significant increase in reepithelialization was seen 14 days post injury. CMHA-S-treated corneas showed significantly less edema than untreated burns and no pathological differences were observed in corneal histological samples [156]. Durrie et al. [157], in patients showed that crosslinked thiolated carboxymethyl HA liquid-gel rapidly reepithelialized the cornea and may promise a well-tolerated and effective therapy for ocular wound care after trauma, disease or surgery.

The first ophthalmic viscosurgical device containing HA was approved by the FDA in 1980 and is still on the market under the trademark Healon (Abbott Medical Optics, IL, USA). Moreover, HA is the active ingredient of many eye drops, such as DropStar by Bracco and Lubristil by Eyelab, which, by hydrating the ocular surface and improving the quality of vision, are efficient in treating diseases such as dry eye syndrome and are useful at increasing the comfortability of contact lenses [83].

### 2.6. Dentistry

Hyaluronic acid is used in dentistry as a biomaterial, since it is the only one with the same chemical structure in all species and tissues. It is additionally used as an adjuvant in tissue reparation and traumatic processes. It is well known that this biopolymer has been employed in treatment of gingivitis, recessions, periodontal pockets due to anti-inflammatory and antibacterial effects and is used as a component of grafts and implants [6,57]. Furthermore, HA can act as a stable barrier against the penetration of viruses and bacteria and elicit a bacteriostatic effect, which are key features to avoid the contamination of surgical wounds by foreign pathogens and to reduce the risk of postoperative infections, thereby promoting a more predictable regeneration including diseases of oral cavity [57]. The results of the study by Pitale et al. [158] showed that the clinical application of injectable HA gel (0.2 mL) for the reconstruction of interdental papillae in 7 patients with 25 defects could be a promising minimally invasive therapy for enhancing papillary esthetics. In another study the in vivo effects of HA gel charged with amino acids (HAplus gel known as Aminogam gel^®^, Errekappa Euroterapici spa, Milan, Italy) and the in vitro effects of the same gel loaded with vitamins C and E to optimize its formulation were examined. This randomized controlled split-mouth clinical and histological trial included 10 adult patients and experiments in vitro were performed in human gingival fibroblast culture. The preliminary results suggested that the use of HAplus gel loaded with vitamins C and E could be beneficial in patients with conditions that impair soft tissue healing [159]. Local applications of metronidazole and 0.2% HA gels as an auxiliary to conventional periodontal therapy have a useful impact on clinical periodontal parameter during 7 days in chronic periodontitis patients [160].

Other applications of HA in dentistry involve: (1) injecting HA for papilla regeneration; (2) covering the dental implant with HA to promote osseointegration; (3) topical application of HA for treatment of oral ulcers; (4) addition of HA to platelet-rich fibrin, plasma and growth factors to improve overall outcomes; (5) using HA as a matrix to encapsulate stem cells and signalling molecules for reconstruction of TMJ, salivary glands, dental pulp, dental bone, enamel, root canal, and mucosa; (6) using HA as a nano-sized drug carrier; and (7) the treatment of stomatitis and irritations caused by fixed or mobile dentures, or during oral surgery procedures [79,161].

In dentistry, HA is commercially used as Hyaloss matrix, which is a product composed of an ester of HA with benzyl alcohol (HYAFF), in a concentration range of 20–60 mg/mL. It is produced as a solid in the form of fibers that form a gel when hydrated, releasing pure HA for approx. 10 days [162]. Ballini et al. [163] showed that Hyaloss matrix in patients can act as a coadjuvant in the grafting processes to produce bone-like tissue in the presence of employing autologous bone obtained from intra-oral sites, to treat infra-bone defects without covering membrane. Moreover, the trials in patients performed by Baldini et al. [164] showed that Hyaloss matrix improved the handling and application of the bone matrix inside the defects and, from a histologic point of view, makes it possible to obtain bone regeneration in less time when it is used with autologous bone.

The next product is Gengigel^®^ (Ricerfarma S.r.l., Milano, Italy), which contains HMM HA in gel formulation at 0.2% concentration for its effect in the treatment of plaque-induced gingivitis as an adjunct to scaling and root planing. The adjunctive use of HA with 0.8% after thorough mechanical debridement potentially has major clinical benefits in terms of improved healing after non-surgical therapy [162]. Gengigel^®^ was effective in conjunction with scaling and root planing may have a beneficial effect on periodontal health in patients with chronic periodontitis [165]. Pistorius et al. [166] found that Gengigel^®^ applied as a spray in patients with gingivitis decreased inflammatory parameters however it did not remove dental plaque. Despite that, the product may be used as an adjuvant in the treatment of gingivitis. One of the challenging and unique periodontal problems of Grade II furcation defects has been managed through different treatment modalities in the past. A successful approach is based on complete closure of the defect. Results of Gupta et al. [167] showed that both Gengigel^®^ with coronally positioned flap and coronally positioned flap alone are effective in the treatment of Grade II furcation defects. The combination of Gengigel^®^ with coronally positioned flap leads to better results in hard tissue measurement as compared to coronally positioned flap alone. It was the first time that a study demonstrated that the topical application of a 0.8% HA gel in the peri-implant pocket and around implants with peri-implantitis may reduce inflammation and crevicular fluid IL-1β levels in patients [168].

### 2.7. Gene Delivery

Gene therapy is the unique way that is able to use the adjustable gene to treat a number of diseases such as inherited disorders, viral infection and various types of cancer. For the gene therapy of human genetic disease gene delivery systems are essentially necessary. For this reason, the aim is to develop clinically relevant vectors such as viral and non-viral vectors that are capable of acting against diseases. Even though this a promising technique in the treatment of incurable diseases, it remains risky and has been under examination so far to make sure that it will be effective safely. The gene therapy has currently been examined only for diseases that have no other cure techniques. Genetic molecules reach the nuclei of host cells to induce gene expression. This therapy has derived to provide a patient’s somatic cells with genetic information for producing specific therapeutic proteins to modulate genetic diseases. In order to get a successful design of gene delivery system, the complete understanding of interaction between targeting cell and gene delivery system is required. The gene delivery systems are composed of the three components such as (a) a gene that encodes a specific therapeutic protein, (b) a plasmid-based gene expression system that controls the function of a gene within the targeting cell, and (c) a gene delivery system that controls the delivery of the gene expression plasmid to specific locations within the body. The appropriate gene delivery system requires the foreign genetic molecule to be stable within the host cells [169,170].

For applications in which transient gene expression is desired, such as tissue regeneration, non-viral vectors offer an attractive choice. Unlike viral vectors, non-viral vectors are characterized by low immunogenicity, ease of production, high nucleic acid packing capacity, high reproducibility and acceptable costs [169,171]. Complementing gene transfer with matrix design for targeted, local DNA delivery has also gained interest in recent years. HA hydrogel scaffolds have been widely studied for their biocompatibility as well as their ability to incorporate a wide variety of molecules, including nucleic acids [171]. A local delivery of DNA through a hydrogel scaffold would increase the applicability of gene therapy in tissue regeneration and cancer therapy. Thereby, a novel process, termed caged nanoparticle encapsulation, has been developed for loading concentrated and unaggregated non-viral gene delivery nanoparticles into various HA hydrogels. Furthermore, HA-based bioconjugates have been developed to enhance selective entry of cytotoxic drugs into HA receptor-expressing cancerous cells (e.g., CD44 and RHAMM in ovarian cancer cells). Results of the study performed by Menaa et al. [24] showed that a new HA-paclitaxel bioconjugate, Oncofid-P, was more potent than paclitaxel alone for intraperitoneal treatment of ovarian cancer in mice. However, HA and its degradation products, accumulated into the stroma of various human tumors, can modulate intracellular signaling pathways and, positively affect angiogenesis of malignant cells and multidrug resistance [24]. Table 3 displays applications of HA in gene therapy.

## 3. Conclusions

This review summarizes properties of HA, which depend on its molar mass. Mention of some knowledge of a rodent—the naked mole rat—is made, which is the object of several investigators worldwide due to the presence of very high HA molar mass. Studies with these animals may help treat cancer in people. Owing to the properties of HA, it has found applications in various fields of medicine. We reported the use of HA in treatment of osteoarthritis and some new studies with HA in the treatment of other joint disorders. Ophthalmology and cosmetics are other fields where HA found a stable position and many commercial preparations containing HA are marketed to date. Several years ago, HA gel found application in dentistry as an adjuvant. Extensive research has been carried out on treatment of difficult-to-heal skin wounds, tissue engineering and gene delivery, where HA alone or with other components had a beneficial effect.

## Figures and Tables

**Table 1:** Wound dressings for treatment of skin wounds composed of HA alone or with another component.

Components	Properties	References
HA	Increased expression of transcripts for the HA receptors CD44, RHAMM, collagen III and I in aged mice	[95]
HA	Reduced inflammation in the wound and promoted skin regeneration compared with the control tests in rabbits	[96]
HA	Alleviated inflammation in the wound, improved skin regeneration and relieved the scar formation in defected skin rabbits	[97]
HA	Accelerated reepithelization, and stimulation of an altered protein expression in human deep dermal incisional skin wounds, no effect on inflammation	[98]
HA-lyophilized fibrin sheet	Higher water retention, faster healing than in untreated animals	[76]
HA-epidermal growth factor (EGF)	More efficient transdermal delivery of HA-EGF conjugates to both normal skin and peripheral tissues around the wound area rather than that of EGF, significantly improved regeneration of skin tissues even in hypodermis of rats	[99]
Modified HA and ε-poly- lysine	Antibacterial effects	[100]
Aminoethyl methacrylate HA	Excellent swelling, mechanical property, low cytotoxicity, rapid hemostasis capacity and facilitated wound healing in mice	[101]
Alginate-HA fibers	Good mechanical performance, high liquid absorption, and swelling percentage, high biocompatibility toward nHDF cell line, maintaining a moist wound surface	[102]
HA-poloxamer	Promoted skin-wound healing and increased protein accumulation in the wound area in rats, higher air permeability than Band-aid	[103]
HA and lysozyme	Hydrogel with suitable viscoelasticity and excellent adhesion on the skin tissue, low cytotoxicity	[104]
γ-Irradiated LMM HA	High viability of L929 skin fibroblasts, faster wound healing after two days of healing in rats	[105]
HA with conjugated azobenzene and β-cyclo-dextrin groups	Hydrogel with fast healing of skin wound in rats	[106]
^●^NO-releasing HA derivatives	Effective against pathogens *Staphylococcus aureus* and *Pseudomonas aeruginosa* in skin wounds, enhanced wound closure and decreased quantity of the *P. aeruginosa* genetic material in the wound tissue in mice wounds	[107]
HA and cellulose	High swelling capacity in various media	[108]
HA and hydroxyethyl cellulose	Appropriate gelation time, good swelling ability, suitable water evaporation rate, well hemocompatibility, biological compatibility, super absorbent capacity	[109]
HA and chitosan	Treatment of skin ulcers, decreased hydration properties of the dressing and modulation of drug release	[82]

**Table 2:** Summary of using HA with other components in engineering of various tissues.

Applications	Components	Properties	References
Soft tissue engineering	Scaffold composed of collagen and HA	Improved resistance to collagenase and elastic mechanical properties, prevention of disintegration of collagen in aqueous medium, cytotoxic for Vero cells	[129]
Articular cartilage tissue engineering	HA and alginate bioink	Improved chondrocyte functionality	[130]
Articular cartilage tissue engineering	Methacrylated gelatin and methacrylated HA	Created cartilage models with varying chondrocyte densities, support of formation of cartilage ECM and recovery of chondrocyte phenotype	[131]
Tissue engineering	UV-crosslinkable HA and surface-modified nanodiamond hydrogels	Mechanically enforced compressive stress, a tunable buffering environment and facilitated interaction with long HA chains at molecular level	[132]
Articular cartilage tissue engineering	HA, hydroxyethyl acrylate and gelatin-methacryloyl bioprinting gel	Stable rheology properties and excellent biocompatibility, viability of bone cells in bioinks of the lattice-printed scaffolds	[133]
Articular cartilage tissue engineering	Silk-HA scaffold	Good biocompatibility with bone marrow mesenchymal stem cells, enhanced cellular proliferation, biodegradable, biomimetic nanofibrous structure	[134]
Bone tissue engineering	Hydrogel composed of arginine-based unsaturated poly(esteramide) and methacrylated HA	Better bone regeneration and expression of osteogenesis-related factors in rats	[135]
Vocal fold tissue engineering	Thiolated HA crosslinked with poly(ethylenglycol) diacrylate (PEGDA) HA-PEGDA with collagen types I or III or their combination	Increased mechanical properties of the hydrogels and reduced hyaluronidase degradation of HA and hydrogel swelling ratio, higher fibroblasts cell adhesion and spreading, viability of cells and synthesized new DNA through 21 days of culture	[136]
Tissue engineering	HA and halloysite nanotubes cryogel as scaffolds	Nonhemolytic materials, macroporous structure, high thermal and mechanical stability, good compatibility, template for cell proliferation, adhesion and the growth media	[137]
Tissue engineering	Carboxymethyl chitosan and partially oxidized HA hydrogel	Moderate swelling, good biocompatibility with embedded cells, sufficient viscosity for printing with good shape fidelity and structural integrity to retain the printed shape	[138]
Bone tissue engineering	Methacrylated HA gels	Good primary chondrocyte survival, excellent spontaneous osteogenic differentiation in vitro	[139]
Bone and cartilage tissue engineering	HA-polyethylenglycol hydrogel	High mechanical stability, enhanced metabolic activity and cell propagation	[140]
Bone and cartilage tissue engineering	HA with methacrylated glycol chitosan hydrogel	Increased propagation and extra deposition of cartilaginous ECM	[141]
Cartilage tissue engineering	Water-based polyurethane based photosensitive materials with HA	Nontoxic, high printing resolution, good cytocompatibility, facilitated chondrocyte adhesion, proliferation and differentiation	[142]
Cartilage tissue engineering	HA-adipic dihydrazide and the oligopeptide grafted oxidized pectin hydrogel	Facilitated chondrogenesis, tissue compatibility by using a mouse subcutaneous implantation model	[143]
Heart valve tissue engineering	Collagen type I and HA scaffold	Mimics fibrosa layer in the aortic valve leaflet ECM, potential to be developed into the trilayer structure of the leaflet, mimics the entire aortic root, cell material system for valve repair	[144]
Soft tissue engineering	Aerogel sponges of silk, fibroin, HA and heparin	High swelling degree and porosity; lower biodegradation; adequate mean pore diameter and connectivity for cells and a soft texture close to that of the brain	[145]
Periodontal tissue engineering	Chitosan-HA scaffold	Increased viability of NIH3T3 and MG63 cell lines, high CD44 expression, physico-chemical parameters without significant morphology changes	[146]
Bone tissue engineering	HA/corn nanosilver-silk β-tricalcium phosphate hydrogel	Antibacterial activity, mesenchymal stem cells high bone differentiation, enhanced physical, mechanical properties, cell binding affinity and tissue compatibility	[147]
Bone tissue engineering	Collagen/HA oligosaccharides/hydroxyapatite and chitosan/HA oligosaccharides in d,l-lactic-co-glycolic acid solution	Stimulation of osteogenesis and endothelialization, promotion of the attachment of endothelial cells, promotion of osteogenic differentiation of MC3T3-E1 and BMSCs cells, ideal biocompatibility and tissue regenerative capacity	[148]
Salivary gland tissue engineering	HA-catechol conjugates	Enhanced cell adhesion, vascular endothelial and progenitor cell proliferation, and branching of cultured embryonic submandibular glands in vitro	[149]
Abdominal tissue regeneration	Chitosan/HA hydrogel	In rats, sufficient extracellular matrix exposition and marked neovascularization were found compared to the control group	[150]

**Table 3:** Application of HA in gene therapy.

Applications	Properties	References
Formation of plasmid DNA (pDNA) powders with LMM HA in pulmonary gene therapy	The highest gene expression in mice, the lactate dehydrogenase activity and concentration of inflammatory cytokines in bronchoalveolar lavage fluid comparable to those caused by ultrapure water	[172]
Transferrin/HA-pDNA/nanostructured lipid carriers in lung cancer gene therapy	Low cytotoxicity, enhanced gene transfer ability in vitro and in vivo	[169]
HA in cochlear gene therapy	Facilitated expression in cells lining the scala media, atraumatic and clinically feasible method to introduce transgenes into cochlear cells	[173]
pDNA/HA/chitosan complexes	Augmented stability and cellular transfection ability of the complexes after lyophilization-rehydration	[174]
Nanoparticles formed by HA, polyethyleneglycol (HA, PEG) and polyethylenimine (HA, PEI) and pDNA	The maximum gene expression using combination of HA, PEG and PEI, negligible cytotoxicity in HeLa and A549 cancer cell lines	[175]
Nanoporous HA hydrogels (NP-HA), annealed HA microparticles (HA-MP) and nanoporous HA hydrogels containing protease-degradable PEG microparticles	Cell densities in scaffolds, distances into which cells penetrated scaffolds and transgene expression levels significantly increased with delivery from HA-MP compared to NP-HA and PEG-MP scaffolds	[176]
Chondroitin sulfate and HA-incorporated sorbitan ester nanoparticles with pDNA	Long-term stability of the nanosystems in both liquid and lyophilized states, viability of A549 cell line, innocuous safety profile in vivo	[177]
Gene delivery to B16F10 cell line by modified HA	Higher gene transfection cytotoxicity, enhanced cellular uptake by HA receptor over-expressed carcinoma cells	[178]
HA modified cationic niosomes (HA-C-niosomes) in retinal gene delivery	Remarkable transfection efficiency in retinal pigment epithelium cells, specified targeting of HA-C-niosomes) in rats	[179]
Oleoyl-carboxymethyl-chitosan (OCMCS)/HA in oral gene vaccine delivery	OCMCS-HA/aerolysis gene polyplexes might resolve challenges arising from gastrointestinal damage to gene antigens	[180]
Micelles conjugated with HA possessing CD44 targeting potential for gene delivery	Prevention of erythrocytes agglutination, high selectivity of the transfection of HMM HA conjugated micelles to cancer cells	[181]
HA and modified chondroitin sulfate in cancer gene delivery	High selectivity of CD44-positive U87 cancer cells and low cytotoxicity in L929 normal cells	[182]
pDNA, lithocholic acid-polyethyleneimine conjugate and HA for gene transfection efficiency	Prepared in different ratios and tested in cells and tumor bearing mice for gene transfer efficiency	[183]
Delivery of DNA from HA-collagen hydrogels	Transgene expression mediated by immobilized, large diameter complexes was 3 to 7-fold greater than for small diameter complexes, greater percentage of cells expressing the transgene for small diameter complexes than for large diameter complexes	[184]
Concentrated nioplexes loaded into HA hydrogels for non-viral gene delivery	Suitable mechanical properties, little or no particle aggregation, efficient transfect mouse bone marrow cloned mesenchymal stem cells in 3D cultures	[171]
HA-shielded polyethylenimine/ pDNA nanogels Gene delivery into human mesenchymal stem cells	Easily internalization of nanogels to human mesenchymal stem cells (HMSC), easy internalized by HeLa cells, increased chondrogenesis of HMSC	[185]
HA microspheres incorporated with DNA for gene delivery or conjugated with an antigen for cell-specific targeting	Sustained release of the encapsulated pDNA for months, transfection in vitro and in vivo. HA microspheres, conjugated with monoclonal antibodies to E- and P-selectin—selective binding to cells expressing these receptors	[186]
Chitosan-HA polyplexes for gene delivery	Compared with chitosan alone, the transfection efficiency had a 4-fold improvement after addition of HA	[187]
HA-chitosan nanoparticles for ocular gene delivery	High transfection levels (up to 15% of cells transfected) without affecting cell viability, internalized by fluid endocytosis	[188]

## Data Availability

The data presented in this study is openly available.
